# Supplementary material for: Changes in Phenotypic and Molecular Features of Naïve and Central Memory T Helper Cell Subsets following SARS-CoV-2 Vaccination
Source: Vaccines (Basel). 2024 Sep 11;12(9):1040. doi: 10.3390/vaccines12091040 (PMC11435719; doi:10.3390/vaccines12091040)
Supplement: Supplementary file 1 [file vaccines-12-01040-s001.zip › vaccines-3162705-supplementary.pdf]

Changes in phenotypic and molecular features of naïve and central memory T helper cell subsets following SARS-CoV-2 vaccination – Supplementary

Author list;

Mia Mosavie[1], Jennifer Rynne[1,2], Matthew Fish[2], Peter Smith[1], Aislinn Jennings[2], Shivani Singh[3], Jonathan Millar[1], Heli Harvala[4], Ana Mora[5], Fotini Kaloyirou[6], Alex Griffiths[7|], Valerie Hopkins[6], Charlotte Washington[8], Lise Estcourt[4], David Robert[4], Manu Shankar-Hari[1]

Affiliations

[1] Centre for Inflammation Research, Institute for Regeneration and Repair, University of Edinburgh, 4-5 Little France Drive, Edinburgh, UK

[2] Department of Transplantation Immunology & Mucosal Biology, School of Immunology and Microbial Sciences, King's College London, London, UK

[3] Department of General Medicine, Charing Cross Hospital, Imperial College Healthcare NHS Trust, London, UK

[4] NHS Blood and Transplant, Colindale, London, UK

[5] HLRI Clinical Research Facility Operations Manager, Cambridge

[6] Statistics and Clinical Research, NHS Blood and Transplant, Cambridge

[7] Statistics and Clinical Research, NHS Blood and Transplant, Bristol

[8] Donor Medicine, NHS Blood and Transplant, Birmingham

Corresponding author

Dr Mia Mosavie PhD

Postdoctoral Research Fellow

Centre for Inflammation Research, Institute for Regeneration and Repair 47 Little France Crescent, Edinburgh, Scotland, UK, EH16 4TJ

Email: mmosavie@ed.ac.uk

ORCiD: https://orcid.org/0000-0002-8332-5948

Funding

This study was supported by National Institute for Health (UKRIDHSC COVID- 19 Rapid Response Rolling Call, “The use of convalescent plasma to treat hospitalized and critically ill patients with COVID-19 disease”, Grant Refer- ence Number COV19-RECPLAS). MF was supported by a National Institute of Academic Anesthesia BJA/RCoA fellowship (WKRO-2018–0047). JR was supported by the Marshall Scholarship and Clarendon Fund. MS-H was funded by a clinician scientist fellowship 2016-16-011 from the National Institute for Health Research (2017-2023).

Disclaimer

The funders/sponsors had no role in the design and conduct of the trial; collection, management, analysis, and interpretation of the data; preparation, review, or approval of the manuscript; and decision to submit the manuscript for publication. The views expressed in this publication are those of the authors and not necessarily those of the National Health Service, the National Institute for Health Research, or the Department of Health and Social Care.

Conflicts of interest

LE reported receiving grants from the National Institute for Health Research and European Union Horizon 2020. MSH reports receiving grant from the Chief Scientists Office, Scotland, for time- critical precision medicine in adult critically ill patients (TRAITS Programme - https://traits-trial.ed.ac.uk).

Supplementary Methods

DNA isolation and ATAC-sequencing

Library preparation was conducted according to the protocol by Buenrostro et al. (2015)13 with adjustments according to the Omni protocol14.
The following buffers were prepared:
Resuspension buffer: 10 mM Tris-HCl pH 7.5, 10 mM NaCl, 3 mM MgCl2.
Lysis buffer: Resuspension buffer plus 0.1 % NP-40, 0.1 % Tween-20, 0.01 % Digi-tonin.
Wash buffer: Resuspension buffer plus 0.1 % Tween-20

50,000 cells were centrifuged at 500 g for 5 minutes at 4 °C, washed with ice-cold PBS and centrifuged at 500 g for 5 minutes at 4°C. The cell pellet was resuspended in 50 µl of ice-cold lysis buffer and incubated on ice for 3 minutes. 1 ml of wash buffer was added, tube was gently inverted three times and centrifuged at 500 g for 10 minutes at 4 °C. Supernatant was discarded and nuclei pellet was gently resuspended in 50 µl of the Transposition reaction mix (25 µl 2X Tagment DNA buffer, 16.5 µl PBS, 0.5 µl 10 % Tween-20 (final 0.1 %), 0.5 µl 1 % digitonin (final 0.01 %), 2.5 µl Tn5 Transposase enzyme, 5 µl nuclease-free water). Transposition mix was incubated at 37 °C for 30 minutes on a thermomixer at 1000 RPM. DNA was isolated using the Qiagen MinElute Reaction Cleanup Kit and eluted in 10 µl nuclease-free water.

PCR amplification of the transposed DNA was performed using the NEBNext High-Fidelity 2X PCR Master Mix and 2.5 µl of each i5 and i7 primer from Nextera XT Index Kit i5 (Illumina) for a final reaction volume of 50 µl with the following PCR specifications; 1 cycle of 72 °C for 5 minutes, 98 °C for 30 seconds, 5 cycles of 98 °C for 10 seconds, 63 °C for 30 seconds, 72 °C for 1 minute. Unique i5 and i7 combinations were used. Quantitative PCR was performed on 5 µl of the partially-amplified libraries to determine the additional PCR cycles required. The following reagents were combined; 5 µl partially-amplified library, 3.85 µl nuclease-free water, 0.5 µl i5 primer 0.5 µl i7 primer, 0.15 µl 100X SYBR Green I, 5 µl NEBNext High-Fidelity 2X PCR Master Mix and qPCR cycles as follows; 1 cycle of 98 °C for 30 seconds, 20 cycles of 98 °C for 10 seconds, 63 °C for 30 seconds, 72 °C for 1 minute. The additional cycle number was calculated for each in-dividual sample by plotting R number versus cycle number to determine the number of cycles needed to reach 1/3 of the maximum fluorescent intensity. The remaining par-tially-amplified library was amplified according to the appropriate cycle number under the following cycle conditions; 1 cycle of 98 °C for 30 seconds, X cycles of 98 °C for 10 seconds, 63 °C for 30 seconds, 72 °C for 1 minute.

AMPure XP beads were used for library purification. Double-sided bead purification was implemented. Beads (0.5 x) were added to each library, mixed thoroughly and in-cubated at room temperature for 10 minutes. Magnetic isolation was performed, the supernatant was transferred to a new tube and 1.3 x original volume AMPure XP beads was added resulting in a final 1.8 x bead buffer: sample ratio, mixed thoroughly, and incubated at room temperature for 10 minutes. Magnetic isolation was performed, and the supernatant was discarded. 80 % ethanol was pipetted over the beads 10 times while on the magnetic rack to wash the beads. The tube was left on the magnetic rack for 10 minutes to dry the beads, ensuring all the ethanol was removed. The beads were resuspended in 20 µl nuclease-free water and placed on the magnetic rack. Purified libraries contained in the supernatant were transferred to a new tube and library quality and quantity was assessed by High Sensitivity D1000 Tapestation (Agilent) and Qubit fluorometer (Thermo Fisher Scientific) respectively.

Libraries were sequenced by Genewiz (Azenta Life Sciences) on a NovaSeq S4 lane (Illumina) platform in 2 × 150 bp configuration to a depth of 50 M paired-end reads.

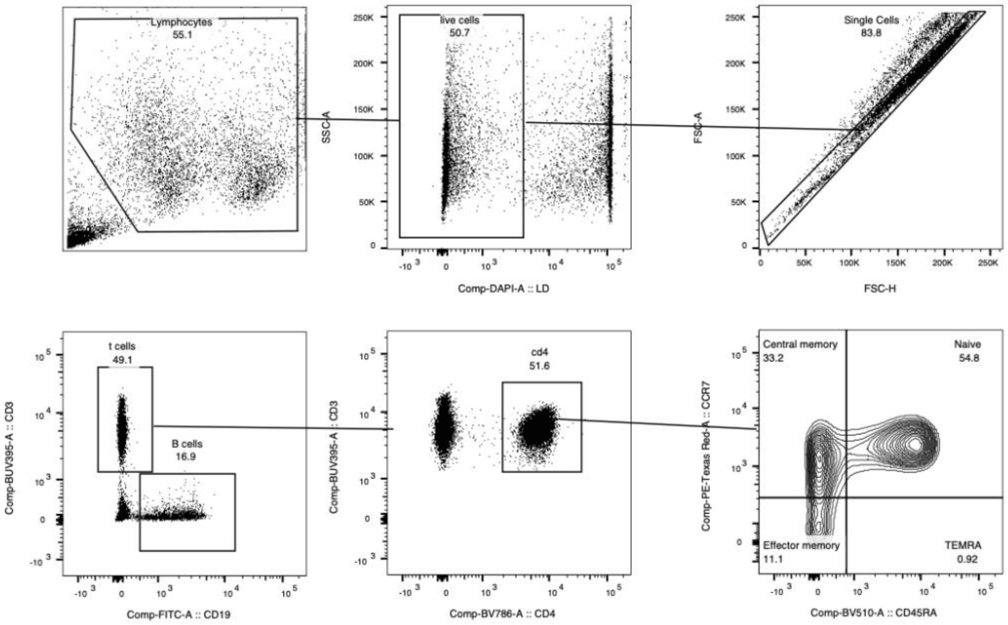

Supplementary Figure 1 – Gating strategy for sorting.

Lymphocytes were sorted into live/dead cells using DAPI, the live cells were then sorted by single cells using the forward scatter gating strategy. T cells were fractioned from B cells, using CD3 and CD19, and $CD4^+$ was fractioned from $CD8^+$ T cells, and finally $CD4^+$naïve T cells were fractioned from $CD4^+$ CM T cells by CCR7 and CD45RA.

Comparisons of molecular features in naïve and central memory T helper cell subsets

Epigenome:

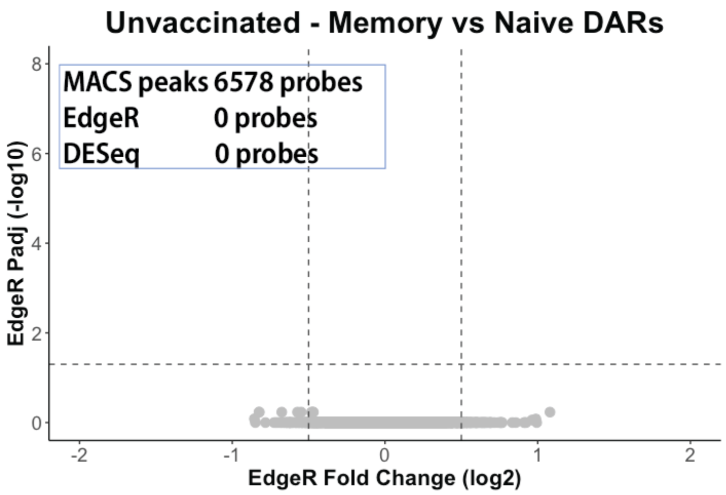

Supplementary Figure 2 - Differentially accessible regions of CD4$^+$CM compared with CD4$^+$naïve in the unvaccinated group.

No DARS found in the unvaccinated group using EdgeR p<0.05 statistical analysis.

Supplementary Table 1 – Top 25 clones of memory vs naïve in TCRA and TCRB, grouped by unvaccinated, one dose vaccinated, two dose vaccinated.

	CDR3.aa	Clones	Proportion	Subset	Vaccine	Status
TCRA	Top 25 clones in Unvaccinated Memory vs Naïve					
1	CAPSGNTPLVF	6	0.000416457	Naive	N	Naive_unvax
2	CAPTGGDTDKLIF	4	0.000250344	Naive	N	Naive_unvax
3	CAVNSGGYQKVTF	17	0.001295477	Naive	N	Naive_unvax
4	CAVNTDKLIF	9	0.000822872	Naive	N	Naive_unvax
5	CAVNTGGFKTIF	24	0.001832791	Naive	N	Naive_unvax
6	CAVNTNAGKSTF	20	0.001660656	Naive	N	Naive_unvax
7	CASRANAGGTSYGKLTF	22	0.007066334	Memory	N	Memory_unvax
8	CAVNTDKLIF	39	0.005486852	Memory	N	Memory_unvax
9	CASPEVTNFGNEKLTF	103	0.005179002	Memory	N	Memory_unvax
10	CAVNTGGFKTIF	43	0.004580375	Memory	N	Memory_unvax
11	CAYLLNAGGTSYGKLTF	11	0.003624382	Memory	N	Memory_unvax
12	CAGAEGNNRKLIW	10	0.003294893	Memory	N	Memory_unvax
13	CALGPDTGGFKTIF	10	0.003294893	Memory	N	Memory_unvax
14	CAERGSNYKLTF	62	0.003117458	Memory	N	Memory_unvax
15	CAYRSPTGNQFYF	60	0.003113672	Memory	N	Memory_unvax
16	CATPVGGFKTIF	9	0.002965404	Memory	N	Memory_unvax
17	CAVNTNAGKSTF	20	0.002720178	Memory	N	Memory_unvax
18	CAVQMIKAAGNKLTF	20	0.002661344	Memory	N	Memory_unvax
19	CAAGGVDDKIIF	8	0.002635914	Memory	N	Memory_unvax
20	CAASIRNFGNEKLTF	8	0.002635914	Memory	N	Memory_unvax
21	CAPSGNTPLVF	9	0.002489774	Memory	N	Memory_unvax
22	CALGDSGTYKYIF	9	0.002376137	Memory	N	Memory_unvax
23	CAGAESSYDKVIF	7	0.002306425	Memory	N	Memory_unvax
24	CAGEKTGFQKLVF	7	0.002306425	Memory	N	Memory_unvax
25	CLVGDMRSGGYQKVTF	7	0.002306425	Memory	N	Memory_unvax
26	CVVSAGGKLIF	7	0.002306425	Memory	N	Memory_unvax
27	CAPTGGDTDKLIF	45	0.002262671	Memory	N	Memory_unvax
28	CAASTGNQGGKLIF	15	0.002205882	Memory	N	Memory_unvax
29	CAVNSGGYQKVTF	12	0.002145262	Memory	N	Memory_unvax
30	CAANGNQFYF	6	0.001976936	Memory	N	Memory_unvax
31	CAARLYNFNKFYF	6	0.001976936	Memory	N	Memory_unvax
	Top 25 clones in One Dose Memory vs Naïve					
1	CAARGAQKLVF	2	0.00014453	Naive	Y	Naive_onedose
2	CAASGGSNYKLTF	15	0.001584505	Naive	Y	Naive_onedose
3	CAENSGGSNYKLTF	35	0.003504167	Naive	Y	Naive_onedose
4	CAFMKVAHDMNRDDKIIF	2	0.000132301	Naive	Y	Naive_onedose
5	CALRRGSQGNLIF	1	0.000138485	Naive	Y	Naive_onedose
6	CAMSRDGQGGKLIF	1	0.000101286	Naive	Y	Naive_onedose
7	CANAGGTSYGKLTF	3	0.000303859	Naive	Y	Naive_onedose
8	CAVDTGRRALTF	22	0.002031806	Naive	Y	Naive_onedose
9	CAVEDQAGTALIF	5	0.00051376	Naive	Y	Naive_onedose
10	CAVNDYKLSF	15	0.001470711	Naive	Y	Naive_onedose
11	CAVNQAGTALIF	28	0.003219879	Naive	Y	Naive_onedose
12	CAVNTGFQKLVF	15	0.00145594	Naive	Y	Naive_onedose
13	CAVNTGGFKTIF	25	0.002458174	Naive	Y	Naive_onedose
14	CAVNTGNQFYF	32	0.002859401	Naive	Y	Naive_onedose
15	CAYADSGNTPLVF	2	0.00027697	Naive	Y	Naive_onedose
16	CAYRSAGGNTPLVF	1	7.2264778147	Naive	Y	Naive_onedose
17	CAYADSGNTPLVF	113	0.015534781	Memory	Y	Memory_onedose
18	CALRDSGGSNYKLTF	84	0.006375712	Memory	Y	Memory_onedose
19	CAFMKVAHDMNRDDKIIF	72	0.005464896	Memory	Y	Memory_onedose
20	CAFMNDFGNEKLTF	64	0.004857685	Memory	Y	Memory_onedose
21	CALRRGSQGNLIF	31	0.004261754	Memory	Y	Memory_onedose
22	CAYYDFGNEKLTF	54	0.004098672	Memory	Y	Memory_onedose
23	CAVDTGRRALTF	29	0.003662404	Memory	Y	Memory_onedose
24	CAVNTGNQFYF	36	0.003435713	Memory	Y	Memory_onedose
25	CAVRPYSGGSNYKLTF	23	0.003161947	Memory	Y	Memory_onedose
26	CAVEDYGGSQGNLIF	24	0.003100058	Memory	Y	Memory_onedose
27	CAYRSAGGNTPLVF	37	0.003082306	Memory	Y	Memory_onedose
28	CAASGGSNYKLTF	25	0.003041126	Memory	Y	Memory_onedose
29	CAVEDQAGTALIF	28	0.003018702	Memory	Y	Memory_onedose
30	CAVQYSSFQKLVF	32	0.002804312	Memory	Y	Memory_onedose
31	CAVNDYKLSF	33	0.002712961	Memory	Y	Memory_onedose
32	CAVNTGFQKLVF	27	0.002577327	Memory	Y	Memory_onedose
33	CANAGGTSYGKLTF	13	0.00250203	Memory	Y	Memory_onedose
34	CAENSGGSNYKLTF	24	0.00250033	Memory	Y	Memory_onedose
35	EGGGNKLTF	18	0.002474567	Memory	Y	Memory_onedose
36	CAARGAQKLVF	22	0.002346231	Memory	Y	Memory_onedose
37	CAVNTGGFKTIF	20	0.002244915	Memory	Y	Memory_onedose
38	CAVNQAGTALIF	21	0.002224026	Memory	Y	Memory_onedose
39	CAVSSTPLVF	10	0.002205347	Memory	Y	Memory_onedose
40	CALDGIQGAQKLVF	16	0.002199615	Memory	Y	Memory_onedose
41	CAMSRDGQGGKLIF	25	0.002190868	Memory	Y	Memory_onedose
	Top 25 clones in Two Dose Memory vs Naïve					
1	CAASANFGNEKLTF	9	0.000821418	Naive	Y	Naive_twodose
2	CAENSGGSNYKLTF	44	0.004177892	Naive	Y	Naive_twodose
3	CAGPNAGNNRKLIW	7	0.000745394	Naive	Y	Naive_twodose
4	CAPGGSQGNLIF	3	0.000258443	Naive	Y	Naive_twodose
5	CATDARGAQKLVF	5	0.000628806	Naive	Y	Naive_twodose
6	CATGGGATNKLIF	2	0.000208961	Naive	Y	Naive_twodose
7	CAVITGGGNKLTF	5	0.000579312	Naive	Y	Naive_twodose
8	CAVKAAGNKLTF	19	0.001643057	Naive	Y	Naive_twodose
9	CAVNDYKLSF	16	0.001540756	Naive	Y	Naive_twodose
10	CAVNQAGTALIF	15	0.001750179	Naive	Y	Naive_twodose
11	CAVNSGGYQKVTF	22	0.002451767	Naive	Y	Naive_twodose
12	CAVNSGNTPLVF	23	0.002512035	Naive	Y	Naive_twodose
13	CAVNTGFQKLVF	5	0.0005782	Naive	Y	Naive_twodose
14	CAVPNQAGTALIF	18	0.001730276	Naive	Y	Naive_twodose
15	CAVQARSGGSNYKLTF	11	0.001418549	Naive	Y	Naive_twodose
16	CAVQASGTYKYIF	10	0.001307646	Naive	Y	Naive_twodose
17	CAVRDDKIIF	12	0.001413441	Naive	Y	Naive_twodose
18	CAVRSTGNQFYF	5	0.000479475	Naive	Y	Naive_twodose
19	CAVTGGFKTIF	4	0.000279558	Naive	Y	Naive_twodose
20	CAVTGNQFYF	32	0.002863178	Naive	Y	Naive_twodose
21	CAVPNQAGTALIF	61	0.005575729	Memory	Y	Memory_twodose
22	CAAVKTSYDKVIF	43	0.004049306	Memory	Y	Memory_twodose
23	CAVNTGFQKLVF	44	0.003876217	Memory	Y	Memory_twodose
24	CAYKADKLIF	26	0.003131398	Memory	Y	Memory_twodose
25	CAENSGGSNYKLTF	39	0.003128792	Memory	Y	Memory_twodose
26	CAVGANAGKSTF	22	0.00303532	Memory	Y	Memory_twodose
27	CAVITGGGNKLTF	12	0.003017802	Memory	Y	Memory_twodose
28	CAVNDYKLSF	25	0.002973212	Memory	Y	Memory_twodose
29	CATDARGAQKLVF	25	0.002938472	Memory	Y	Memory_twodose
30	CAVRSTGNQFYF	25	0.002888974	Memory	Y	Memory_twodose
31	CATGGGATNKLIF	23	0.002770083	Memory	Y	Memory_twodose
32	CAESRRRALTF	30	0.002712968	Memory	Y	Memory_twodose
33	CAVTGNQFYF	30	0.002687011	Memory	Y	Memory_twodose
34	CAVNSGNTPLVF	27	0.002684775	Memory	Y	Memory_twodose
35	CAVKAAGNKLTF	27	0.002570799	Memory	Y	Memory_twodose
36	CAGPNAGNNRKLIW	15	0.002549461	Memory	Y	Memory_twodose
37	CAARGDDMRF	32	0.002531646	Memory	Y	Memory_twodose
38	CAVQASGTYKYIF	30	0.002508962	Memory	Y	Memory_twodose
39	CAVTGGFKTIF	21	0.00245352	Memory	Y	Memory_twodose
40	CAPGGSQGNLIF	16	0.00244497	Memory	Y	Memory_twodose
41	CAVQARSGGSNYKLTF	29	0.002431687	Memory	Y	Memory_twodose
42	CAVNQAGTALIF	24	0.002361569	Memory	Y	Memory_twodose
43	CAASANFGNEKLTF	23	0.002244133	Memory	Y	Memory_twodose
44	CAVNSGGYQKVTF	22	0.002243754	Memory	Y	Memory_twodose
45	CAVRDDKIIF	14	0.002224745	Memory	Y	Memory_twodose

	CDR3.aa	Clones	Proportion	Subset	Vaccine	Status
TCRB	Top 25 clones in Unvaccinated Memory vs Naive					
1	CASNIGGNGGELFF	1	2.27159147698878e-05	Naive	N	Naive_unvax
2	CASSFEGGGGEQFF	1	2.76388159531246e-05	Naive	N	Naive_unvax
3	CASSLGGGTGKKNF	1	2.27159147698878e-05	Naive	N	Naive_unvax
4	CASSLGGVNNEQFF	4	0.000110555	Naive	N	Naive_unvax
5	CASSPGQGQTQYF	2	4.54318295397756e-05	Naive	N	Naive_unvax
6	CASSPLAGVDTQYF	1	2.76388159531246e-05	Naive	N	Naive_unvax
7	CASSSGSTDTQYF	9	0.000213869	Naive	N	Naive_unvax
8	CASSSRVADNEQFF	1	2.27159147698878e-05	Naive	N	Naive_unvax
9	CASTLRAMWPYRGWYGELFF	3	6.81477443096634e-05	Naive	N	Naive_unvax
10	CSARDHGLAGADTQYF	1	2.27159147698878e-05	Naive	N	Naive_unvax
11	CASSSGSTDTQYF	292	0.006835763	Memory	N	Memory_unvax
12	CSANPGTGGGNEQFF	139	0.003249258	Memory	N	Memory_unvax
13	CASNIGGNGGELFF	121	0.002828491	Memory	N	Memory_unvax
14	CASSSNRGRGKTQYF	116	0.002711611	Memory	N	Memory_unvax
15	CASSSRVADNEQFF	110	0.002571355	Memory	N	Memory_unvax
16	CATSDDSGGRVTGELFF	48	0.002386042	Memory	N	Memory_unvax
17	CASSLGGGTGKKNF	100	0.002337596	Memory	N	Memory_unvax
18	CASSFEGGGGEQFF	47	0.002336332	Memory	N	Memory_unvax
19	CSVAPAASGSYEQYF	46	0.002236593	Memory	N	Memory_unvax
20	CASSLESGTEQFF	43	0.002137496	Memory	N	Memory_unvax
21	CASSPSGTLYEQYF	40	0.001988368	Memory	N	Memory_unvax
22	CASSEMKGGTDTQYF	40	0.001944863	Memory	N	Memory_unvax
23	CAISELANTGELFF	53	0.001768081	Memory	N	Memory_unvax
24	CASSLGGVNNEQFF	35	0.001739822	Memory	N	Memory_unvax
25	CASSQVFSAGTSGSQPVGTQYF	52	0.001734721	Memory	N	Memory_unvax
26	CSARDHGLAGADTQYF	74	0.001729821	Memory	N	Memory_unvax
27	CASTLRAMWPYRGWYGELFF	72	0.001683069	Memory	N	Memory_unvax
28	CASSLRLAGPGNEQFF	50	0.001668001	Memory	N	Memory_unvax
29	CASTLNGAKNIQYF	69	0.001612941	Memory	N	Memory_unvax
30	CASSPGQGQTQYF	69	0.001612941	Memory	N	Memory_unvax
31	CASSPLAGVDTQYF	47	0.001567921	Memory	N	Memory_unvax
32	CASNHRDGYEQYF	66	0.001542813	Memory	N	Memory_unvax
33	CASSVTQIGEFTEAFF	45	0.001501201	Memory	N	Memory_unvax
34	CASSSPSGGWQFF	62	0.001449309	Memory	N	Memory_unvax
35	CASSPGQGATNEQFF	29	0.001441567	Memory	N	Memory_unvax
	Top 25 clones in One Dose Memory vs Naïve					
1	CASSHREGYEQYF	3	5.80787547914973e-05	Naive	Y	Naive_onedose
2	CASSLAGSYNEQFF	6	0.000132788	Naive	Y	Naive_onedose
3	CASSLRQGIAKNIQYF	2	3.87191698609982e-05	Naive	Y	Naive_onedose
4	CASSLVPDGNYEQYF	5	8.73530285294991e-05	Naive	Y	Naive_onedose
5	CASSPDTYNEQFF	2	6.23994097217578e-05	Naive	Y	Naive_onedose
6	CASSRDRSSYEQYF	4	0.000120061	Naive	Y	Naive_onedose
7	CASSRRLGTSGRGQETQYF	2	3.87191698609982e-05	Naive	Y	Naive_onedose
8	CASSSSGGADTQYF	2	5.50344751323633e-05	Naive	Y	Naive_onedose
9	CSAPDRTDTQYF	2	6.96252425259391e-05	Naive	Y	Naive_onedose
10	CSASQSNEQFF	5	9.67979246524955e-05	Naive	Y	Naive_onedose
11	CASSLGDRGSNGELFF	346	0.00958316	Memory	Y	Memory_onedose
12	CASSHREGYEQYF	316	0.009273389	Memory	Y	Memory_onedose
13	CASSPMDLRGEQYF	304	0.008419886	Memory	Y	Memory_onedose
14	CSARFNNYGYTF	184	0.005096247	Memory	Y	Memory_onedose
15	CSASQSNEQFF	154	0.00451931	Memory	Y	Memory_onedose
16	CASSRDRSSYEQYF	130	0.004252674	Memory	Y	Memory_onedose
17	CASRSPGQGADEQFF	151	0.004182246	Memory	Y	Memory_onedose
18	CASSRGQASGALNTGELFF	139	0.004079117	Memory	Y	Memory_onedose
19	CASSTGGSEDYEQFF	191	0.003329034	Memory	Y	Memory_onedose
20	CSAPDRTDTQYF	87	0.00284602	Memory	Y	Memory_onedose
21	CASSSSGGADTQYF	142	0.002708528	Memory	Y	Memory_onedose
22	CASSPDTYNEQFF	81	0.002649743	Memory	Y	Memory_onedose
23	CASSRRLGTSGRGQETQYF	81	0.00237704	Memory	Y	Memory_onedose
24	CASSLAVGGGNTEAFF	19	0.002327861	Memory	Y	Memory_onedose
25	CASSFGQDYEQYF	82	0.002271154	Memory	Y	Memory_onedose
26	CASSTSGISTDTQYF	18	0.002205342	Memory	Y	Memory_onedose
27	CASSLAGSYNEQFF	78	0.002139146	Memory	Y	Memory_onedose
28	CASSSTVGGGNEQFF	104	0.001983711	Memory	Y	Memory_onedose
29	CASSTGTGGDTQYF	103	0.001964637	Memory	Y	Memory_onedose
30	CASSLDGSGPYEQYF	60	0.001962773	Memory	Y	Memory_onedose
31	CASTIKGVGNQPQHF	16	0.001960304	Memory	Y	Memory_onedose
32	CASSLRQGIAKNIQYF	66	0.001936847	Memory	Y	Memory_onedose
33	CASSLVPDGNYEQYF	100	0.001907414	Memory	Y	Memory_onedose
34	CSVLSGGEETQYF	106	0.001847527	Memory	Y	Memory_onedose
35	CASSSGGLASQETQYF	66	0.001828002	Memory	Y	Memory_onedose
	Top 25 clones in Two Dose Memory vs Naive					
1	CASRSQETQYF	2	7.47328301322771e-05	Naive	Y	Naive_two
2	CASSFNSGANVLTF	6	0.000136752	Naive	Y	Naive_two
3	CASSLAGTDTQYF	12	0.000260203	Naive	Y	Naive_two
4	CASSLRLAGAEEAFF	4	9.11680911680912e-05	Naive	Y	Naive_two
5	CASSLYGARYEQYF	20	0.000302371	Naive	Y	Naive_two
6	CASSPQGEKLFF	1	2.27920227920228e-05	Naive	Y	Naive_two
7	CASSPTSGTYNEQFF	3	6.72147494589856e-05	Naive	Y	Naive_two
8	CASSQRGASSYEQYF	6	0.000139876	Naive	Y	Naive_two
9	CSVESTGGETQYF	1	3.73664150661386e-05	Naive	Y	Naive_two
10	CASSLDSNYGYTF	118	0.003537417	Memory	Y	Memory_twodose
11	CSASPTSRSTDTQYF	93	0.003436807	Memory	Y	Memory_twodose
12	CASSLRLAGAEEAFF	90	0.003325942	Memory	Y	Memory_twodose
13	CASSRAGTNTGELFF	94	0.003130842	Memory	Y	Memory_twodose
14	CASSLYGARYEQYF	91	0.002730027	Memory	Y	Memory_twodose
15	CAISETRTGGTGELFF	74	0.002472436	Memory	Y	Memory_twodose
16	CASSLAGTDTQYF	91	0.0020861	Memory	Y	Memory_twodose
17	CASSPDRGYEQYF	62	0.0020715	Memory	Y	Memory_twodose
18	CASSLNSGPTHEQYF	52	0.001921656	Memory	Y	Memory_twodose
19	CSVQSGSSYEQYF	56	0.001871032	Memory	Y	Memory_twodose
20	CSVLRGAEETQYF	79	0.001863735	Memory	Y	Memory_twodose
21	CASSLGTGPYNEQFF	78	0.001846552	Memory	Y	Memory_twodose
22	CASSGGLAGVNEQFF	48	0.001760473	Memory	Y	Memory_twodose
23	CASSRFLGSGANVLTF	73	0.001722186	Memory	Y	Memory_twodose
24	CASSPPPRAGTTDTQYF	57	0.001710017	Memory	Y	Memory_twodose
25	CASSPQGEKLFF	51	0.001703976	Memory	Y	Memory_twodose
26	CASSHSTGGTYEQYF	61	0.001699354	Memory	Y	Memory_twodose
27	CASSFNSGANVLTF	45	0.001662971	Memory	Y	Memory_twodose
28	CSVESTGGETQYF	49	0.001637153	Memory	Y	Memory_twodose
29	CASSQSRELFF	35	0.001626394	Memory	Y	Memory_twodose
30	CASSPHRTVNTEAFF	34	0.001579926	Memory	Y	Memory_twodose
31	CASSQRGASSYEQYF	85	0.001519539	Memory	Y	Memory_twodose
32	CASSPTSGTYNEQFF	41	0.001515152	Memory	Y	Memory_twodose
33	CASRSQETQYF	54	0.001504346	Memory	Y	Memory_twodose
34	CSARDRQLAKNIQYF	54	0.001504346	Memory	Y	Memory_twodose

Supplementary Table 2 - TCR matched epitopes grouped by unvaccinated, one dose vaccinated, two dose vaccinated and overall vaccinated (combined one dose and two dose)

Trimmed input sequence	Match sequence	Score	Receptor group	Epitope	Antigen	Organism
**UNVACCINATED**						
ASSSGSTDTQY	ASSGGSTDTQY	0.97017	88336	NLVPMVATV,GLEAPFLYLYALVYFLQSI NFVRIIMR	HCMVUL83,ORF3a protein	Human herpesvirus 5 (Human cytomegalovirus),SARS-CoV2
ASSSGSTDTQY	ASSEGSTDTQY	0.97145	10157	NLVPMVATV	HCMVUL83	Human herpesvirus 5 (Human cytomegalovirus)
ASSSGSTDTQY	ASRSGSTDTQY	0.97116	53135	GILGFVFTL	Matrix protein 1	Influenza A virus
ASSSGSTDTQY	ASSGSTDTQY	0.97574	147697	MIELSLIDFYLCFLAFLLFLVLIML	ORF7b	SARS-CoV2
ASSSGSTDTQY	ASSGSTDTQY	0.97574	87493	CALDPLSETK	surface glycoprotein	SARS-CoV2
ASSSGSTDTQY	ASSSSGSTDTQY	0.9787	123527	YLDAYNMMI	orf1ab polyprotein	SARS-CoV2
ASSSGSTDTQY	ASTSGSTDTQY	0.9848	134958	HTTDPSFLGRY	orf1ab polyprotein	SARS-CoV2
ASSREAQETQY	ASREAQETQY	0.97176	32543	STLPETAVVRR	precore/core protein	Hepatitis B virus (Human hepatitis B virus)
SARQQGYEQY	SAREQGYEQY	0.9742	142279	MIELSLIDFYLCFLAFLLFLVLIML	ORF7b	SARS-CoV2
SASLGGADTQY	ASSLGGADTQY	0.98583	199993	EAAGIGILTV,NLSALGIFST	Melanoma antigen recognized by T-cells 1,Insulin-like growth factor 2 mRNA-binding protein 2	Homo sapiens (human),Homo sapiens (human)
SASLGGADTQY	ASSLGGADTQY	0.98583	92858	QLMCQPILLL,HTTDPSFLGRY	orf1ab polprotein,orf1ab polyprotein	SARS-CoV2,SARS-CoV2
ASSLAGTENQPQH	ASSVAGTENQPQH	0.97931	195867	GILGFVFTL,LLLDRLNQL	Matrix protein 1,Nucleoprotein	Influenza A virus,SARS coronavirus BJ01
ASSLEGTDTQY	ASSLEGTDTQY	1	93532	FVCNLLLLFVTVYSHLLLV	ORF3a protein	SARS-CoV2
ASSLEGTDTQY	ASSLKGTDTQY	0.97001	97200	VPHVGEIPVAYRKVLL	orf1ab polyprotein	SARS-CoV2
ASSLERNTEAF	ASSLDRNTEAF	0.97061	200086	FLAHIQWMV,VSALSRAAEK	orf1ab polyprotein	SARS-CoV2
ASSLQGSTEAF	ASSIQGSTEAF	0.98392	40429	AVFDRKSDAK,KLGGALQAK	Epstein-Barr nuclear antigen 4,55 kDa immediate-early protein 1	Human herpesvirus 4 (Epstein Barr virus),Human herpesvirus 5 strain AD169 (Human cytomegalovirus (strain AD169))
ASSLQGSTEAF	ASSLQGATEAF	0.97953	54235	KLGGALQAK	55 kDa immediate-early protein 1	Human herpesvirus 5 strain AD169 (Human cytomegalovirus (strain AD169))
**ONE DOSE**						
SASQSNEQF	ASSQANEQF	0.97078	17545	GILGFVFTL	Matrix protein 1	Influenza A virus
SASQSNEQF	ASSQSNEQF	0.98684	122016	FLWLLWPVTLACFVLAAV	membrane glycoprotein	SARS-CoV2
SASQSNEQF	ASTQSNEQF	0.97176	134039	FLWLLWPVTLACFVLAAV	membrane glycoprotein	SARS-CoV2
ASSRDRSSYEQY	ASSRDRSSYEQY	1	36641	RAKFKQLL	Lytic switch protein BZLF1	Human herpesvirus 4 (Epstein Barr virus)
ASSRDRSSYEQY	ASGRDRSSYEQY	0.97092	70746	NPLLYDANYFLCW	ORF3a protein	SARS-CoV2
ASSSSGGADTQY	ASSASGGADTQY	0.98287	37318	KLGGALQAK	55 kDa immediate-early protein 1	Human herpesvirus 5 strain AD169 (Human cytomegalovirus (strain AD169))
ASSSSGGADTQY	ASSSSGGSDTQY	0.98135	49759	KLGGALQAK	55 kDa immediate-early protein 1	Human herpesvirus 5 strain AD169 (Human cytomegalovirus (strain AD169))
ASSSSGGADTQY	ASSSGGADTQY	0.97647	120927	RQLLFVVEV,KAYNVTQAF	orf1ab polyprotein ,nucleocapsid phosphoprotein	SARS-CoV2,SARS-CoV2
ASSSSGGADTQY	ASSSGGADTQY	0.97647	187374	KYNKANVFL		
ASSSSGGADTQY	ASSSTGGADTQY	0.98034	124860	AELAKNVSLDNVL,MIELSLIDFYLCFLAF LLFLVLIML	orf1ab polyprotein,ORF7b	SARS-CoV2,SARS-CoV2
ASSPDTYNEQF	ASSPDSYNEQF	0.97444	179634	KLGGALQAK,APHGVVFLHVTYV,VGAT YYFNKNMSTYVDYKIN	55 kDa immediate-early protein 1,surface glycoprotein,porin OmpC [Escherichia coli]	Human herpesvirus 5 strain AD169 (Human cytomegalovirus (strain AD169)),SARS-CoV2,Escherichia coli
ASSFGQDYEQY	ASSYGQDYEQY	0.97293	130912	KPFERDISTEIY	surface glycoprotein	SARS-CoV2
ASSTSGISTDTQY	ASSSTGISTDTQY	0.97459	123316	FLNGSCGSV	orf1ab polyprotein	SARS-CoV2
ASSTSGISTDTQY	ASSSSGISTDTQY	0.98301	123388	FLNGSCGSV	orf1ab polyprotein	SARS-CoV2
ASSTSGISTDTQY	ASSSSGVSTDTQY	0.97384	123726	FLNGSCGSV,MPASWVMRI	orf1ab polyprotein,orf1ab polyprotein	SARS-CoV2,SARS-CoV2
ASSTSGISTDTQY	ASSTTGISTDTQY	0.98107	128858	FLNGSCGSV	orf1ab polyprotein	SARS-CoV2
ASSTSGISTDTQY	ASSTSGVATDTQY	0.97039	130182	VPHVGEIPVAYRKVLL	orf1ab polyprotein	SARS-CoV2
ASSTSGISTDTQY	ASSTSGVSTDTQY	0.99009	130183	FLNGSCGSV	orf1ab polyprotein	SARS-CoV2
ASSLAGSYNEQF	ASSLAGAYNEQF	0.97728	88944	NLVPMVATV,VLWAHGFEL,VPHVGEIP VAYRKVLL,YEQYIKWPWYI	HCMVUL83,orf1ab polyprotein,orf1ab polyprotein,surface glycoprotein	Human herpesvirus 5 (Human cytomegalovirus),SARS-CoV2,SARS-CoV2,SARS-CoV2
ASSLAGSYNEQF	ASSLSGSYNEQF	0.97879	99946	GILGFVFTL,VQPTESIVRFPNITNLCPF	Matrix protein 1,surface glycoprotein	Influenza A virus,SARS-CoV2
ASSLAGSYNEQF	ASNLAGSYNEQF	0.97539	73603	FLWLLWPVTLACFVLAAV	membrane glycoprotein	SARS-CoV2
ASSLAGSYNEQF	ASSIAGSYNEQF	0.98342	85957	VLWAHGFEL	orf1ab polyprotein	SARS-CoV2
ASSLAGSYNEQF	ASSLAGSYNEQF	1	170759	APHGVVFLHVTYV,YEQYIKWPWYI,AVF DRKSDAK	surface glycoprotein,surface glycoprotein,Epstein-Barr nuclear antigen 4	SARS-CoV2,SARS-CoV2,Human herpesvirus 4 (Epstein Barr virus)
ASSLAGSYNEQF	ASSLAGTYNEQF	0.9762	90042	IMLIIFWFSL,VLWAHGFEL	ORF7b,orf1ab polyprotein	SARS-CoV2,SARS-CoV2
ASSLAGSYNEQF	ASSVAGSYNEQF	0.97954	190275	LVQSTQWSL	Replicase polyprotein 1ab	SARS-CoV2
**TWO DOSE**						
ASSLDSNYGYT	ASSLDSNYGYT	1	94624	MIELSLIDFYLCFLAFLLFLVLIML	ORF7b	SARS-CoV2
ASSLDSNYGYT	ASSLDANYGYT	0.97621	95009	MIELSLIDFYLCFLAFLLFLVLIML	ORF7b	SARS-CoV2
ASSRAGTNTGELF	ASSRSGTNTGELF	0.9791	120630	ILLIIMRTFKVSIWNLDYII	ORF6 protein	SARS-CoV2
ASSLAGTDTQY	ASSLSGTDTQY	0.97933	54204	KLGGALQAK	55 kDa immediate-early protein 1	Human herpesvirus 5 strain AD169 (Human cytomegalovirus (strain AD169))
ASSLAGTDTQY	ASSLAGTDTQY	1	90132	QLMCQPILLL	orf1ab polyprotein	SARS-CoV2
ASSLAGTDTQY	ASSLAGTETQY	0.97355	90139	VQPTESIVRFPNITNLCPF	surface glycoprotein	SARS-CoV2
ASSPDRGYEQY	ASSPDRGYEQY	1	108320	MIELSLIDFYLCFLAFLLFLVLIML	ORF7b	SARS-CoV2
ASSLGTGPYNEQF	ASSLGTGPYNEQF	1	20849	GILGFVFTL	Matrix protein 1	Influenza A virus
ASSLGTGPYNEQF	ASSLGTGPYNEQF	1	96198	GILGFVFTL,GNYTVSCLPFTI	Matrix protein 1,ORF8 protein	Influenza A virus,SARS-CoV2
ASSLGTGPYNEQF	ASSLGTGPYNEQF	1	20849	GILGFVFTL	Matrix protein 1	Influenza A virus
ASSLGTGPYNEQF	ASSLGTGPYNEQF	1	96198	GILGFVFTL,GNYTVSCLPFTI	Matrix protein 1,ORF8 protein	Influenza A virus,SARS-CoV2
ASSFNSGANVLT	ASSFSSGANVLT	0.97008	12921	NLVPMVATV	HCMVUL83	Human herpesvirus 5 (Human cytomegalovirus)
ASSFNSGANVLT	ASSFNSGANVLT	1	82449	FCNDPFLGVYY	surface glycoprotein	SARS-CoV2
ASSFNSGANVLT	ASSLNSGANVLT	0.97077	101393	AYSNNSIAIPTNFTISV	surface glycoprotein	SARS-CoV2
ASSQRGASSYEQY	ASSRRGASSYEQY	0.97226	120195	APKEIIFLEGETL	orf1ab polyprotein	SARS-CoV2
ASSPTSGTYNEQF	ASSPTSGSYNEQF	0.97552	115267	HTTDPSFLGRY	orf1ab polyprotein	SARS-CoV2
ASRSQETQY	ASRSEETQY	0.97579	21563	NLVPMVATV	HCMVUL83	Human herpesvirus 5 (Human cytomegalovirus)
ASRSQETQY	ASSRSQETQY	0.97244	172423	GLCTLVAML	Transcriptional regulator IE63 homolog	Human herpesvirus 4 (Epstein Barr virus)
**VACCINATED**						
SASQSNEQF	ASSQANEQF	0.97078	17545	GILGFVFTL	Matrix protein 1	Influenza A virus
SASQSNEQF	ASSQSNEQF	0.98684	122016	FLWLLWPVTLACFVLAAV	membrane glycoprotein	SARS-CoV2
SASQSNEQF	ASTQSNEQF	0.97176	134039	FLWLLWPVTLACFVLAAV	membrane glycoprotein	SARS-CoV2
ASSRDRSSYEQY	ASSRDRSSYEQY	1	36641	RAKFKQLL	Lytic switch protein BZLF1	Human herpesvirus 4 (Epstein Barr virus)
ASSRDRSSYEQY	ASGRDRSSYEQY	0.97092	70746	NPLLYDANYFLCW	ORF3a protein	SARS-CoV2
ASSSSGGADTQY	ASSASGGADTQY	0.98287	37318	KLGGALQAK	55 kDa immediate-early protein 1	Human herpesvirus 5 strain AD169 (Human cytomegalovirus (strain AD169))
ASSSSGGADTQY	ASSSSGGSDTQY	0.98135	49759	KLGGALQAK	55 kDa immediate-early protein 1	Human herpesvirus 5 strain AD169 (Human cytomegalovirus (strain AD169))
ASSSSGGADTQY	ASSSGGADTQY	0.97647	120927	RQLLFVVEV,KAYNVTQAF	orf1ab polyprotein,nucleocapsid phosphoprotein	SARS-CoV2,SARS-CoV2
ASSSSGGADTQY	ASSSGGADTQY	0.97647	187374	KYNKANVFL		
ASSSSGGADTQY	ASSSTGGADTQY	0.98034	124860	AELAKNVSLDNVL,MIELSLIDFYLCFLAF LLFLVLIML	orf1ab polyprotein,ORF7b	SARS-CoV2,SARS-CoV2
ASSPDTYNEQF	ASSPDSYNEQF	0.97444	179634	KLGGALQAK,APHGVVFLHVTYV,VGAT YYFNKNMSTYVDYKIN	55 kDa immediate-early protein 1,surface glycoprotein,porin OmpC [Escherichia coli]	Human herpesvirus 5 strain AD169 (Human cytomegalovirus (strain AD169)),SARS-CoV2,Escherichia coli
ASSFGQDYEQY	ASSYGQDYEQY	0.97293	130912	KPFERDISTEIY	surface glycoprotein	SARS-CoV2
ASSTSGISTDTQY	ASSSTGISTDTQY	0.97459	123316	FLNGSCGSV	orf1ab polyprotein	SARS-CoV2
ASSTSGISTDTQY	ASSSSGISTDTQY	0.98301	123388	FLNGSCGSV	orf1ab polyprotein	SARS-CoV2
ASSTSGISTDTQY	ASSSSGVSTDTQY	0.97384	123726	FLNGSCGSV,MPASWVMRI	orf1ab polyprotein,orf1ab polyprotein	SARS-CoV2,SARS-CoV2
ASSTSGISTDTQY	ASSTTGISTDTQY	0.98107	128858	FLNGSCGSV	orf1ab polyprotein	SARS-CoV2
ASSTSGISTDTQY	ASSTSGVATDTQY	0.97039	130182	VPHVGEIPVAYRKVLL	orf1ab polyprotein	SARS-CoV2
ASSTSGISTDTQY	ASSTSGVSTDTQY	0.99009	130183	FLNGSCGSV	orf1ab polyprotein	SARS-CoV2
ASSLAGSYNEQF	ASSLAGAYNEQF	0.97728	88944	NLVPMVATV,VLWAHGFEL,VPHVGEIP VAYRKVLL,YEQYIKWPWYI	HCMVUL83,orf1ab polyprotein,orf1ab polyprotein,surface glycoprotein	Human herpesvirus 5 (Human cytomegalovirus),SARS-CoV2,SARS-CoV2,SARS-CoV2
ASSLAGSYNEQF	ASSLSGSYNEQF	0.97879	99946	GILGFVFTL,VQPTESIVRFPNITNLCPF	Matrix protein 1,surface glycoprotein	Influenza A virus,SARS-CoV2
ASSLAGSYNEQF	ASNLAGSYNEQF	0.97539	73603	FLWLLWPVTLACFVLAAV	membrane glycoprotein	SARS-CoV2
ASSLAGSYNEQF	ASSIAGSYNEQF	0.98342	85957	VLWAHGFEL	orf1ab polyprotein	SARS-CoV2
ASSLAGSYNEQF	ASSLAGSYNEQF	1	170759	APHGVVFLHVTYV,YEQYIKWPWYI,AVF DRKSDAK	surface glycoprotein,surface glycoprotein,Epstein-Barr nuclear antigen 4	SARS-CoV2,SARS-CoV2,Human herpesvirus 4 (Epstein Barr virus)
ASSLAGSYNEQF	ASSLAGTYNEQF	0.9762	90042	IMLIIFWFSL,VLWAHGFEL	ORF7b,orf1ab polyprotein	SARS-CoV2,SARS-CoV2
ASSLAGSYNEQF	ASSVAGSYNEQF	0.97954	190275	LVQSTQWSL	Replicase polyprotein 1ab	SARS-CoV2
ASSLDSNYGYT	ASSLDSNYGYT	1	94624	MIELSLIDFYLCFLAFLLFLVLIML	ORF7b	SARS-CoV2
ASSLDSNYGYT	ASSLDANYGYT	0.97621	95009	MIELSLIDFYLCFLAFLLFLVLIML	ORF7b	SARS-CoV2
ASSRAGTNTGELF	ASSRSGTNTGELF	0.9791	120630	ILLIIMRTFKVSIWNLDYII	ORF6 protein	SARS-CoV2
ASSLAGTDTQY	ASSLSGTDTQY	0.97933	54204	KLGGALQAK	55 kDa immediate-early protein 1	Human herpesvirus 5 strain AD169 (Human cytomegalovirus (strain AD169))
ASSLAGTDTQY	ASSLAGTDTQY	1	90132	QLMCQPILLL	orf1ab polyprotein	SARS-CoV2
ASSLAGTDTQY	ASSLAGTETQY	0.97355	90139	VQPTESIVRFPNITNLCPF	surface glycoprotein	SARS-CoV2
ASSPDRGYEQY	ASSPDRGYEQY	1	108320	MIELSLIDFYLCFLAFLLFLVLIML	ORF7b	SARS-CoV2
ASSLGTGPYNEQF	ASSLGTGPYNEQF	1	20849	GILGFVFTL	Matrix protein 1	Influenza A virus
ASSLGTGPYNEQF	ASSLGTGPYNEQF	1	96198	GILGFVFTL,GNYTVSCLPFTI	Matrix protein 1,ORF8 protein	Influenza A virus,SARS-CoV2
ASSLGTGPYNEQF	ASSLGTGPYNEQF	1	20849	GILGFVFTL	Matrix protein 1	Influenza A virus
ASSLGTGPYNEQF	ASSLGTGPYNEQF	1	96198	GILGFVFTL,GNYTVSCLPFTI	Matrix protein 1,ORF8 protein	Influenza A virus,SARS-CoV2
ASSFNSGANVLT	ASSFSSGANVLT	0.97008	12921	NLVPMVATV	HCMVUL83	Human herpesvirus 5 (Human cytomegalovirus)
ASSFNSGANVLT	ASSFNSGANVLT	1	82449	FCNDPFLGVYY	surface glycoprotein	SARS-CoV2
ASSFNSGANVLT	ASSLNSGANVLT	0.97077	101393	AYSNNSIAIPTNFTISV	surface glycoprotein	SARS-CoV2
ASSQRGASSYEQY	ASSRRGASSYEQY	0.97226	120195	APKEIIFLEGETL	orf1ab polyprotein	SARS-CoV2
ASSPTSGTYNEQF	ASSPTSGSYNEQF	0.97552	115267	HTTDPSFLGRY	orf1ab polyprotein	SARS-CoV2
ASRSQETQY	ASRSEETQY	0.97579	21563	NLVPMVATV	HCMVUL83	Human herpesvirus 5 (Human cytomegalovirus)
ASRSQETQY	ASSRSQETQY	0.97244	172423	GLCTLVAML	Transcriptional regulator IE63 homolog	Human herpesvirus 4 (Epstein Barr virus)

Comparisons of repertoire features between unvaccinated, single dose vaccinated, two dose vaccinated and combined vaccinated groups

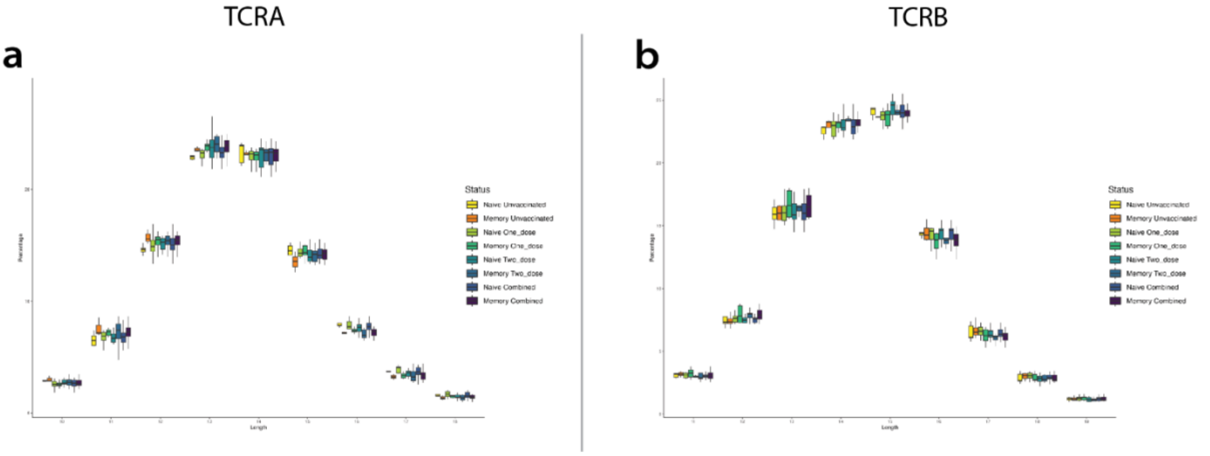

Supplementary Figure 3 – cell repertoire changes in naïve and memory subsets.

Amino acid length of CDR3 in **a)** TCRα and **b)** TCRβ chains. The total number of unique TCR sequences per participant was used to determine the percentage of cells with each CDR3 length. CDR3 lengths that did not occur were not included in the visualisation.
